# WeChat-based mHealth intention and preferences among people living with schizophrenia

**DOI:** 10.7717/peerj.10550

**Published:** 2020-12-16

**Authors:** Shuiyuan Xiao, Tongxin Li, Wei Zhou, Minxue Shen, Yu Yu

**Affiliations:** 1 Department of Social Medicine and Health Management, Xiangya School of Public Health, Central South University, Changsha, Hunan, China; 2 School of Public Administration, Hunan University, Changsha, Hunan, China; 3 Division of Prevention and Community Research, Department of Psychiatry, Yale School of Medicine, New Haven, CT, United States of America

**Keywords:** Schizophrenia, WeChat, mHealth, Intention, Preferences, Chinese

## Abstract

**Background:**

The past few decades have seen a rapid expansion of mHealth programs among people with serious mental illness, yet mHealth for schizophrenia is in a much earlier stage of development. This study examined the intention of WeChat-based mHealth programs among people living with schizophrenia (PLS) and evaluated correlates of the intention.

**Methods:**

A total of 400 PLS aged 18–77 completed a cross-sectional survey by face-to-face interviews. The survey included a general question asking about participants’ willingness to attend WeChat-based mHealth programs, followed by preferences of three specific WeChat-based programs: psychoeducation, peer support, and professional support. PLS symptoms, functioning and disability were measured using the 18-item Brief Psychiatric Rating Scale (BPRS-18), the Global Assessment of Functioning (GAF), and the 12-item World Health Organization Disability Assessment Schedule 2.0 (WHODAS 2.0), respectively. A multivariate logistic regression was used to determine correlates of program participation intention.

**Results:**

Over forty percent (43%, *n* = 172) of participants were willing to participate in WeChat-based mHealth programs, among whom preferences for each specific program were shown in descending order: psychoeducation (68.60%), professional support (60.47%), and peer support (52.33%). A multivariate analysis revealed that younger age (OR: 0.13–0.20, 95% CI [0.05–0.43]), higher education (OR: 3.48–6.84, 95% CI [1.69–18.21]), and lower disability (OR: 0.97, 95% CI [0.94–0.99]) were all independently associated with WeChat-based mHealth program participation intention.

**Conclusion:**

The findings provide guidance for further development of WeChat-based mHealth programs among PLS in China, and targeted at those who are younger, well-educated and with lower disability.

## Introduction

Schizophrenia represents a major public health problem globally that affects over 21 million people worldwide and accounts for huge global burden of disease (Charlson et al., 2018; WHO, 2010). Schizophrenia has a significant impact on individuals, families, and society since it results in elevated risk of relapse, rehospitalization, incarceration, poverty, homelessness, substance use, early mortality, and suicide (Draine et al., 2002; Liu et al., 2017). However, many people living with schizophrenia (PLS) do not receive adequate care or receive poor quality care (Kazdin, 2017). Reasons for the treatment gap may include time and financial burden, poor availability of services (e.g., long distance from psychiatric hospital/clinic, transportation), stigma associated with seeking care, and dissatisfaction with services (McGuire et al., 2016; Mojtabai et al., 2009). The huge disease burden and significant treatment gap of schizophrenia have driven the advancement of mobile health (mHealth) that use mobile phones in support of health care to complement for the traditional clinical-based care (Farrell, Mahone & Guilbaud, 2004).

Mobile health (mHealth) can be defined as the use of mobile computing and communication technologies in health care and public health (Free et al., 2010). Due to their characteristics of accessibility, portability, affordability, online-connectivity, and ease of use (Alvarez-Jimenez et al., 2012), mobile phones are ubiquitous, even among people with serious mental illness who often have limited access to resources (Ben-Zeev et al., 2013; Naslund, Aschbrenner & Bartels, 2016). Research showed a mobile phone use rate of over 80% among PLS and people with other psychosis (Firth et al., 2016). During the past few years, there is an exponential increase in the development of mHealth strategies that provide non-stigmatizing and easily-assessible support to PLS (Alvarez-Jimenez et al., 2013). These have included, for example, mobile apps to assess functioning and symptoms (Ben-Zeev et al., 2014), on-line peer support groups and forums to exchange information and share emotions (Haker, Lauber & Rossler, 2005), web-based psychoeducation and psychological therapies to increase knowledge and promote recovery (Arnberg et al., 2014), and wearable technologies that offer real-time feedback on well-being and functioning (Cella et al., 2018). So far, promising results have been shown for the acceptability, feasibility and efficiency of mHealth in mental health promotion and health care delivery in the world (Kannisto, Koivunen & Valimaki, 2014; Lindhiem et al., 2015; Sahu, Grover & Joshi, 2014).

In China, the most dominant mobile app is WeChat (literally: micro message) owned by Chinese company tech giant Tencent (Pang, 2018). First released in January 2011, WeChat quickly gathered huge momentum and popularity, and has become the most widely and extensively used mobile social networking application in China (Zhang et al., 2017). The wide application of WeChat into every human being’s daily life indicates a promising new medium for delivering healthcare in a cost-effective way. Accumulating evidence has robustly shown that WeChat-based mHealth is acceptable, feasible, and cost-effective in improving health outcomes among various health conditions (Feng et al., 2017; Liu et al., 2018; Wang et al., 2019).

Despite the high infiltration of mHealth in the world, and the wide popularity of WeChat use in China, to date there is a paucity of research on WeChat-based health interventions among PLS in China. A growing body of research indicates that people with serious mental illness are increasingly turning to social media platforms to connect to individuals with similar conditions and to obtain health-related information (Khazaal et al., 2008). A recent meta-analysis on mobile phone ownership and endorsement of “mHealth” among people with psychosis has shown that over 80% of PLS and people with other psychoses use mobile phone on a routine basis, and mHealth interventions were endorsed by a majority of them (Firth et al., 2016). However, whether this holds true for WeChat-based health interventions among PLS in China remains unknown. The current study was conducted to understand intentions and preferences for WeChat-based mHealth in a community sample of PLS in China and determine correlates of participation intention. Our results may contribute to the further development of WeChat-based mHealth programs to deliver targeted needs-based health services for this population.

## Materials & Methods

### Study population

This cross-sectional study was conducted from May 2019 to September 2019 in the Changsha Psychiatric Hospital that provides community-based mental healthcare through the “686 Program”. The 686 Program is China’s largest demonstration project in mental health that provides free services such as physical check, medication delivery, follow-up and emergency hospitalization for all registered people with serious mental illness including schizophrenia (Good & Good, 2012; Ma, 2012). A multistage cluster-sampling method was adopted to get a representative sample of all community residents in Changsha city. First, four districts (Yuelu, Furong, Kaifu, and Tianxin) were randomly selected from nine administrative districts. Second, two to four communities were randomly selected from each district (three for Yuelu, two for Furong, four for Kaifu, and three for Tianxin) depending on its population size and registered number of people with serious mental illness in the 686 Program. Finally, a total sampling frame of 12 communities were selected that were representative of residents in Changsha city, in terms of geography, socio-demographics, mental health care access and outreach activities. Participants were included if they were clients of the “686 Program”; diagnosed with schizophrenia according to the criteria of the Chinese Classification of Mental Disorders-3 (CCMD-3) or the International Classification of Diseases-10 (ICD-10); living with a family member; aged at least 18; and able to read and communicate. They were excluded if they were having other mental illness diagnosis other than schizophrenia such as depression, dementia, epilepsy, or being too mentally disabled or too illiterate to read or communicate.

### Procedures

Ethical approval was granted by the Institutional Review Committee of the Xiangya School of Public Health of Central South University (No.: XYGW-2019-029). Each month, a medical team composed of both psychiatrists and psychiatry nurses from Changsha Psychiatric Hospital will circulate the 12 community health centers to distribute anti-psychotic medicine and provide assessment and consultation to registered participants with serious mental illness. The interviewers were a research team of three psychiatrists from Changsha Psychiatric Hospital who joined the medical team to circulate the 12 health centers to recruit and interview participants. A poster with detailed information of the study was posted in each health center to promote study participation. Each registered client with mental illness that came to the health center for free medicine or consultation was referred by the medical team to the research team. Those who were eligible for the study were then invited to participate in the study. After providing written informed consent, each participant received clinical assessment by the research team of psychiatrists, as well as completed a questionnaire survey, with or without the assistance of family members. The interview took approximately 25-35 minutes, and each participant was reimbursed with 10 RMB ($1.4) in cash for their participation.

### Measures

#### Program intention and preferences

Program intention was assessed by asking participants whether they were willing to participate in WeChat-based mhealth programs. Those who answered “yes” were further asked three “Yes/No” questions assessing their preferences for various programs, including psychoeducation, peer support and professional support. Sample questions include: “Are you willing to participate We-Chat based psychoeducation such as following a WeChat Official Account(WOA) on mental illness to get knowledge and learn skills?”, “Are you willing to join a WeChat peer support group with patients with similar conditions to share experiences and emotions?”, and “Are you willing to add psychiatrists as your WeChat friends to have online consultation and timely interaction?”

#### Symptoms

PLS symptoms were measured by the 18-item Brief Psychiatric Rating Scale (BPRS-18) to assess a set of common symptom characteristics in psychiatric patients (Dazzi, Shafer & Lauriola, 2016). It covers five domains of clinical symptoms: affect, positive symptoms, negative symptoms, resistance, and activation as proposed by Shafer (Shafer, 2005). Each item is rated on an 8-point Likert scale from 0- “not assessed”, 1- “not at all” to 7- “extremely severe”. The total score ranges from 0-126, with higher score representing greater severity of symptoms. The BPRS-18 has been frequently used in schizophrenia with well-established psychometric properties (Dazzi, Shafer & Lauriola, 2016; Shafer, 2005). In the current study, the BPRS-18 showed good internal consistency, with a Cronbach’s alpha of 0.85.

#### Functioning

PLS functioning was assessed by the Global Assessment of Functioning (GAF) to measure a person’s psychological, social, and occupational functioning on a hypothetical continuum of mental health-illness ranging from 1 to 100 (Goldman, Skodol & Lave, 1992). It is a one-item question, with higher score indicating better patient functioning. Examples are given for each ten-level interval. GAF has also been widely used in clinical assessment with satisfactory psychometric properties established (Association, 1994). In the current study we assessed the functional level of individuals living with schizophrenia over the past 1 month.

#### Disability

Disability was assessed by the 12-item World Health Organization Disability Assessment Schedule 2.0 (WHODAS 2.0) (Sheehan, Harnett-Sheehan & Raj, 1996; WHO, 2017) to measure disability and functional impairment. It covers six domains of functioning: cognition, mobility, self-care, getting along with people, life activities, and participation in society (Sheehan, Harnett-Sheehan & Raj, 1996). Each item is rated on a 5-point Likert scale from 0- “no difficulty” to 4- “extreme difficulty” to assess the level of difficulty experienced while performing the activities. The total score ranges from 0-48, with higher score representing higher level of disability. In the current study, the WHODAS 2.0 showed good internal consistency, with a Cronbach’s alpha of 0.89.

### Statistical analysis

Scales and indices were tested for reliability. Exploratory and summary statistics were obtained for all variables within the dataset. Those who were willing to participate in WeChat-based mHealth intervention programs and those who were not were compared for socio-demographics and clinical characteristics by χ^2^ test or *t*-test. A multivariate logistic regression was further conducted to determine correlates of participation intention, with WeChat-based mHealth program intention as dependent variable, and age, gender, marriage, education, occupation, symptoms, functioning, and disability as independent variables. All data were analyzed using STATA version 16. Values of p less than 0.05 were considered statistically significant (two-tailed test).

## Results

### Sample characteristics

A total of 400 participants completed the questionnaire. Participants were equally distributed by gender. The majority were married or cohabited (43%), and in the 46–59 age group (40.75%). Over two thirds have an education level of middle and high school (67.8%), and nearly 90% were unemployed.

### WeChat-based mHealth program intention and preferences

Table 1 presents participants’ intention and preferences for WeChat-based mHealth programs. Among all 400 participants, 172 (43%) indicates willingness to participate in any kind of WeChat-based mHealth programs. Among the three forms of WeChat-based mHealth programs, psychoeducation was the most commonly endorsed one (68.60%), followed by professional support (60.47%), and peer support (52.33%). The majority were willing to participate in only one form of WeChat-based intervention (46.15%), followed by all three forms (27.91%) and two forms only (25.58%).

**Table 1:** WeChat-based mHealth intention and preferences (*N* = 400).

**Variables**	**N**	**%**
Are you willing to participate in WeChat-based mHealth programs? (*N* = 400)		
No	228	57.00
Yes	172	43.00
Are you willing to participate in WeChat-based psychoeducation? (*N* = 172)		
No	54	31.40
Yes	118	68.60
Are you willing to join WeChat-based peer support group? (*N* = 172)		
No	82	47.67
Yes	90	52.33
Are you willing to receive WeChat-based professional support? (*N* = 172)		
No	68	39.53
Yes	104	60.47
Number of WeChat-based mHealth programs that respondents were willing to participate (*N* = 172)		
Only 1	80	46.51
Only 2	44	25.58
All 3	48	27.91

### Comparison of WeChat-based mHealth program supporters and non-supporters

Table 2 shows comparison of socio-demographics (e.g., age, gender, marriage, education, occupation) and clinical characteristics (symptoms, functioning and disability) between those who were willing to participate in WeChat-based mHealth programs and those who were not. Significant differences were found in all variables except for gender and occupation. Compared to those who were not willing to participate in WeChat-based mHealth programs, those who were willing to participate were generally of younger age (18-35 age group: 25% vs 9.65%, *p* < 0.001), with a higher education (college and above: 19.19% vs 9.21%, *p* = 0.025), more likely to be married or cohabited (45.35% vs 41.23%, *p* = 0.025). Furthermore, those who were willing to participate had lower symptom scores (31.35 vs 34.05, *p* = 0.026), higher functioning score (64.50 vs 59.85, *p* = 0.001) and lower disability score (23.70 vs 27.78, *p* < 0.001) than those who were not.

**Table 2:** Social-demographic and clinical characteristics and WeChat-based mHealth program intention (*n* = 400).

**Characteristic**	**All respondents(*n* = 400)**		**WeChat-based mHealth intention**				**p**^b^
			**No (*n* = 228)**		**Yes (*n* = 172)**		
	**N**	**%**	**N**	**%**	**N**	**%**	
**Social-demographic characteristics**							
Gender							
Male	200	50.00	116	50.88	84	48.84	0.686
Female	200	50.00	112	49.12	88	51.16	
Age							
18–35	65	16.25	22	9.65	43	25.00	**<0.001**
36–45	121	30.25	59	25.88	62	36.05	
46–59	163	40.75	108	47.37	55	31.98	
60–100	51	12.75	39	17.11	12	6.98	
Marriage							
Single	150	37.50	79	34.65	71	41.28	**0.025**
Married/cohabited	172	43.00	94	41.23	78	45.35	
Else^a^	78	19.50	55	24.12	23	13.37	
Education							
Primary & below	75	18.75	59	25.88	16	9.30	**0.025**
Middle & high	271	67.75	148	64.91	123	71.51	
College & above	54	13.50	21	9.21	33	19.19	
Occupation							
Unemployed	358	89.50	210	92.11	148	86.05	0.050
Employed	42	10.50	18	7.89	24	13.95	
**Clinical characteristics**							
BPRS-18	32.90 ± 11.43		34.05 ± 0.82		31.35 ± 0.86		**0.026**
GAF	61.83 ±13.58		59.85 ± 0.85		64.50 ± 1.22		**0.001**
WHODAS 2.0	26.02 ±10.22		27.78 ± 0.72		23.70 ± 0.69		**<0.001**

**Notes.**

a Else include divorced, separated, and widowed.

b Descriptive statistics were compared with chi-square tests for categorical variables and *t*-test for continuous variables, values in bold represents significant at *p* < 0.05 or *p* < 0.01.

BPRS-18 18-item Brief Psychiatric Rating Scale GAF Global Assessment of 5 Functioning WHODAS 2.0 World Health Organization Disability Assessment Schedule 2.0

### Predictor of WeChat-based mHealth programs intention

Table 3 shows results of a further multivariate logistic regression to determine the correlates of WeChat-based mHealth programs intention. Among all eight factors that were included in the model, only three remained significant after controlling for all other variables: age, education and disability. Age negatively predicted program intention, with the likelihood of participating in WeChat-based mHealth programs decreasing by 80% in the 46–59 age group (adjusted odds ration (AOR) = 0.20, 95% CI [0.09–0.43]), and 87% in the >60 age group (AOR = 0.13, 95% CI [0.05–0.36]), compared to the 18–35 age group. Education positively predicted program intention. Compared to those with primary and below education, those with middle and high school education, and those with college and above education were 3.48 (AOR = 3.48, 95% CI [1.69–7.17]) and 6.84 (AOR = 6.84, 95% CI [2.57–18.21]) times, respectively, as likely to participate in WeChat-based mHealth programs. Disability also negatively predicted program intention, with the likelihood of participating decreasing by 3% with each one-point increase in disability score (AOR = 0.97, 95% CI [0.94–0.99]).

**Table 3:** Multivariate linear regression of WeChat-based mHealth program intention.

**Variable**		**WeChat use**	
		**aOR**^b^**(95% CI)**	**P**
Age	18–35	ref	
	36–45	0.54 (0.26, 1.13)	0.102
	46–59	0.20 (0.09, 0.43)	**<0.001**
	60–100	0.13 (0.05, 0.36)	**<0.001**
Gender	Male	ref	
	Female	1.00 (0.60, 1.68)	1.00
Marriage	Single	ref	
	Married/cohabited	1.72 (0.93, 3.17)	0.082
	Else^a^	0.83 (0.39, 1.77)	0.627
Education	Primary & below	ref	
	Middle & high	3.48 (1.69, 7.17)	**0.001**
	College & above	6.84 (2.57, 18.21)	**<0.001**
Occupation	Unemployed	ref	
	Employed	1.16 (0.53, 2.54)	0.720
BPRS-18		1.00 (0.97, 1.03)	0.912
GAF		1.01 (0.99, 1.04)	0.350
WHODAS 2.0		0.97 (0.94, 0.99)	**0.023**

**Notes.**

a Values in bold represents significant at *p* < 0.05 or *p* < 0.01.

b aOR: adjusted odds ratio.

BPRS-18 18-item Brief Psychiatric Rating Scale GAF Global Assessment of Functioning WHODAS 2.0 World Health Organization Disability Assessment Schedule 2.0

## Discussion

The finding that 43% of participants were willing to participate in WeChat-based mHealth programs resonates with a WeChat use rate of 40.75% among PLS in the current study. This rate was higher than that reported in other studies about online mental health program through social medias in and out of China (Borzekowski et al., 2009; Kidd et al., 2019; Miller et al., 2015). For instance, Borzekowski et al reported only one third of PLS and people with other serious mental illness used internet for health information (Borzekowski et al., 2009). In Miller et al’s study on social media use among PLS, only 27% of PLS used social networking sites daily (Miller et al., 2015). A careful comparison between WeChat-based mHealth programs in the current study with other social media-based mHealth programs among PLS revealed some advantages of the WeChat-based programs. Compared to other commonly used social media such as Facebook, Twitter, Vblog that serves only the purpose of social networking in other countries, WeChat in China is much more widely popularized in people’s various aspects of daily life for its diverse functioning apart from social communication, such as payment, transportation, taxi, and amusement. Compared to other mHealth programs that focused only on psychoeducation or peer support, our multi-component programs combined various elements of interventions and thus are more welcomed by participants. The promising number shows that it is acceptable to deliver mental health interventions to PLS WeChat users through the WeChat platform. However, what is noteworthy is that there is still a large proportion of PLS population (57%) who were not willing to participate in WeChat-based mHealth programs, implying some potential barriers and challenges lying in such programs for PLS. Several common difficulties have been posited, for instance, some PLS cannot afford a mobile phone due to economic difficulties, some are not able to use mobile phone and WeChat due to poor functioning, some hold negative attitudes towards mHealth due to concern of stigma, bullying, privacy and confidentiality (Naslund & Aschbrenner, 2019; Torous & Keshavan, 2016). This finding indicates that some measures may be taken to increase affordability, usability and safety of WeChat-based mHealth programs. Another implication is that caregivers may also participate in those programs to assist PLS to better utilize such resources, especially when PLS were with poor functioning. In general, the intension rate of 43% for WeChat-based mHealth programs offers optimism in future development and expansion of such programs among PLS, while suggesting room for improvement in getting more PLS involved.

Among the three proposed WeChat-based mHealth program elements, psychoeducation is the most commonly endorsed mode of intervention by the participants. This finding is consistent with the bulk of literature showing psychoeducation as the predominant and most effective intervention to improve well-being of PLS and their families (Khalil et al., 2019; Oksuz et al., 2017). Evidence shows that psycho-education not only significantly decrease relapse and improve recovery in PLS, but also decrease family burden and improve psychosocial well-beings in family members (Pekkala & Merinder, 2002; Sorrell, 2014). One implication may be that future WeChat-based mhealth intervention programs may consider psychoeducation as its core component. For instance, a WeChat Official Account (WOA) that publishes articles and videos to provide education on schizophrenia and training on symptom management may be appropriate.

Apart from psychoeducation, professional support and peer support have also been well accepted by the participants, with over half showing willingness to participate in such programs. These findings also correspond to the robust evidence supporting both professional support and peer support. Accumulating evidence has shown support provided by professional healthcare workers such as psychiatrists and psychiatry nurses promotes a healthy lifestyle among PLS by providing both spiritual encouragement and professional guidance on treatment adherence, which contributes to improved prognosis of PLS (Gandhi et al., 2019; Salzmann-Erikson & Sjodin, 2018). Peer support has also been found to be beneficial in maintaining the psychological and social well-being of PLS, as it allows group members to disclose negative feelings, provide positive emotional support, and share strategies to deal with challenges and difficulties (Chien & Norman, 2009). These findings suggest that future WeChat-based mhealth intervention programs may benefit from adding both professional support and peer support as its supplementary components to maximize intervention effect and promote the well-being of PLS. For instance, PLS may add their psychiatrists as their WeChat friends to provide targeted help and address specific needs. Besides, a WeChat chat group may be set up for PLS to share feelings and experiences, exchange useful tips and information, and obtain mutual support with others in similar conditions.

The finding that both younger age and higher education significantly and independently predicted WeChat-based mHealth program participation intention is in accordance with most previous studies showing higher endorsement of mHealth among those with younger age and higher education (Borzekowski et al., 2009; Villani & Kovess-Masfety, 2017). Compared to old people, young people are more likely to embrace new technology and more willing to try innovative interventions. As a result, they are more likely to accept WeChat-based mHealth programs. Compared to those who are less-educated, those with higher education have higher health literacy, are more capable to navigate through the WeChat platform for information and support, as a result, they are more willing to participate in WeChat-based mHealth program. Another explanation may be that people who are younger and higher-educated may have better access to WeChat and thus are more willing to participate in WeChat-based mHealth programs. One implication of these finding is that future WeChat-based mHealth programs may consider targeting those who are younger and with better education in order to maximize intervention benefits. Another implication is that some basic training on WeChat use may be provided to those who are older and less educated to promote participation rate among PLS.

Regarding disability, lower disability positively predicts the intention to participate in WeChat-based mHealth program. Although not reported in previous study, the negative association between disability and program participation intention can be well explained by the concept and measurement of disability in the current study. Using the 12-item World Health Organization Disability Assessment Schedule 2.0 (WHODAS 2.0) (Sheehan, Harnett-Sheehan & Raj, 1996), we measured the following six domains of functioning: cognition, mobility, self-care, getting along with people, life activities, and participation in society. PLS with higher disability may be too cognitively impaired to use WeChat, or too socially impaired to participate in any WeChat-based mHealth program that involves social contacts and social communication. For instance, some PLS who are highly disabled may have difficulty in recognizing messages sent from WeChat and may misunderstand some friendly greetings as offending remarks and thus refuse to respond or respond inappropriately. On the other hand, PLS with lower disability are more likely to express willingness to participate in WeChat-based mHealth program. This finding has implication for future intervention study to improve disability among PLS to increase their intention to participate in and thus benefit from WeChat-based mHealth programs.

One limitation is that our sample was all recruited from the 686 Program of Changsha city, which may preclude PLS not registered in the 686 Program, and PLS in other parts of China that may be different from PLS in Changsha. Future research may benefit from conducting multi-center community study that includes both registered and non-registered PLS from various areas of China to get a more representative national sample and to use standardized rate to reflect the standardized rate of willingness to use mHealth among PLS in China. Another limitation is the lack of qualitative data on participants’ preferences towards specific WeChat-based mHealth programs to get a deeper understanding of what specific contents participants like and why do they like them. Future study may consider using mixed methods research methods to get both a comprehensive and deeper understanding of participants’ preferences for WeChat-based mHealth programs. A third limitation is that we didn’t include caregivers’ use of WeChat in the current study, considering the essential roles caregivers play in caring for PLS, it would be beneficial for future study to also involve caregivers in WeChat-based mHealth programs. A fourth limitation is that we only focused on WeChat for PLS, it remains unknown whether and how PLS are using other social media both in and out of China. Future research may include other social media such as QQ, Facebook, Twitter, Instagram to make comparisons with WeChat use.

## Conclusions

As far as we know, this is the first study to empirically explore intentions and preferences towards innovative WeChat-based mHealth programs among a Chinese community sample of PLS. Our findings show the possibility and acceptability to deliver mental health interventions to PLS through the WeChat platform. The results also provide the following implications for future WeChat-based mHealth programs: (1) focus on psychoeducation as core component, professional support and peer support as supplementary component; (2) target at specific populations such as those who are younger, well-educated, and with lower disability in order to achieve the best benefits; (3) provide basic training on WeChat use for those who are older and less educated; (4) improve disability of PLS to increase their participation in WeChat-based mHealth programs; (5) involve family caregivers in WeChat-based mHealth programs to assist PLS in better utilizing resources.

##  Supplemental Information

10.7717/peerj.10550/supp-1 Supplemental Information 1 Original dataset

10.7717/peerj.10550/supp-2 Supplemental Information 2 Analytic codes for running the data analysis using STATA

## Additional Information and Declaration

### Data Availability

The following information was supplied regarding data availability: The data and codes are available in the Supplemental Files.

## References

[ref-1] Alvarez-Jimenez M, Bendall S, Lederman R, Wadley G, Chinnery G, Vargas S, Larkin M, Killackey E, McGorry PD, Gleeson JF (2013). On the HORYZON: moderated online social therapy for long-term recovery in first episode psychosis. Schizophrenia Research.

[ref-2] Alvarez-Jimenez M, Gleeson JF, Bendall S, Lederman R, Wadley G, Killackey E, McGorry PD (2012). Internet-based interventions for psychosis: a sneak-peek into the future. Psychiatric Clinics of North America.

[ref-3] Arnberg FK, Linton SJ, Hultcrantz M, Heintz E, Jonsson U (2014). Internet-delivered psychological treatments for mood and anxiety disorders: a systematic review of their efficacy, safety, and cost-effectiveness. PLOS ONE.

[ref-4] American Psychiatric Association (1994). Diagnostic and statistical manual of mental disorders. 4th ed (DSM-IV).

[ref-5] Ben-Zeev D, Brenner CJ, Begale M, Duffecy J, Mohr DC, Mueser KT (2014). Feasibility, acceptability, and preliminary efficacy of a smartphone intervention for schizophrenia. Schizophrenia Bulletin.

[ref-6] Ben-Zeev D, Davis KE, Kaiser S, Krzsos I, Drake RE (2013). Mobile technologies among people with serious mental illness: opportunities for future services. Administration and Policy in Mental Health.

[ref-7] Borzekowski DL, Leith J, Medoff DR, Potts W, Dixon LB, Balis T, Hackman AL, Himelhoch S (2009). Use of the internet and other media for health information among clinic outpatients with serious mental illness. Psychiatric Services.

[ref-8] Cella M, Okruszek L, Lawrence M, Zarlenga V, He Z, Wykes T (2018). Using wearable technology to detect the autonomic signature of illness severity in schizophrenia. Schizophrenia Research.

[ref-9] Charlson FJ, Ferrari AJ, Santomauro DF, Diminic S, Stockings E, Scott JG, McGrath JJ, Whiteford HA (2018). Global epidemiology and burden of schizophrenia: findings from the global burden of disease study 2016. Schizophrenia Bulletin.

[ref-10] Chien WT, Norman I (2009). The effectiveness and active ingredients of mutual support groups for family caregivers of people with psychotic disorders: a literature review. International Journal of Nursing Studies.

[ref-11] Dazzi F, Shafer A, Lauriola M (2016). Meta-analysis of the Brief Psychiatric Rating Scale - Expanded (BPRS-E) structure and arguments for a new version. Journal of Psychiatric Research.

[ref-12] Draine J, Salzer MS, Culhane DP, Hadley TR (2002). Role of social disadvantage in crime, joblessness, and homelessness among persons with serious mental illness. Psychiatric Services.

[ref-13] Farrell SP, Mahone IH, Guilbaud P (2004). Web technology for persons with serious mental illness. Archives of Psychiatric Nursing.

[ref-14] Feng S, Liang Z, Zhang R, Liao W, Chen Y, Fan Y, Li H (2017). Effects of mobile phone WeChat services improve adherence to corticosteroid nasal spray treatment for chronic rhinosinusitis after functional endoscopic sinus surgery: a 3-month follow-up study. European Archives of Oto-Rhino-Laryngology.

[ref-15] Firth J, Cotter J, Torous J, Bucci S, Firth JA, Yung AR (2016). Mobile phone ownership and endorsement of “mHealth” among people with psychosis: a meta-analysis of cross-sectional studies. Schizophrenia Bulletin.

[ref-16] Free C, Phillips G, Felix L, Galli L, Patel V, Edwards P (2010). The effectiveness of M-health technologies for improving health and health services: a systematic review protocol. BMC Research Notes.

[ref-17] Gandhi S, Gurusamy J, Damodharan D, Ganesan V, Palaniappan M (2019). Facilitators of healthy life style behaviors in persons with schizophrenia—a qualitative feasibility pilot study. Asian Journal of Psychiatry.

[ref-18] Goldman HH, Skodol AE, Lave TR (1992). Revising axis V for DSM-IV: a review of measures of social functioning. American Journal of Psychiatry.

[ref-19] Good BJ, Good MJ (2012). Significance of the 686 Program for China and for global mental health. Shanghai Arch Psychiatry.

[ref-20] Haker H, Lauber C, Rossler W (2005). Internet forums: a self-help approach for individuals with schizophrenia?. Acta Psychiatrica Scandinavica.

[ref-21] Kannisto KA, Koivunen MH, Valimaki MA (2014). Use of mobile phone text message reminders in health care services: a narrative literature review. Journal of Medical Internet Research.

[ref-22] Kazdin AE (2017). Addressing the treatment gap: a key challenge for extending evidence-based psychosocial interventions. Behaviour Research and Therapy.

[ref-23] Khalil AH, Nahas GEL, Ramy H, Abdel Aziz K, Elkholy H, El-Ghamry R (2019). Impact of a culturally adapted behavioural family psychoeducational programme in patients with schizophrenia in Egypt. International Journal of Psychiatry in Clinical Practice.

[ref-24] Khazaal Y, Chatton A, Cochand S, Hoch A, Khankarli MB, Khan R, Zullino DF (2008). Internet use by patients with psychiatric disorders in search for general and medical informations. Psychiatric Quarterly.

[ref-25] Kidd SA, Feldcamp L, Adler A, Kaleis L, Wang W, Vichnevetski K, McKenzie K, Voineskos A (2019). Feasibility and outcomes of a multi-function mobile health approach for the schizophrenia spectrum: App4Independence (A4i). PLOS ONE.

[ref-26] Lindhiem O, Bennett CB, Rosen D, Silk J (2015). Mobile technology boosts the effectiveness of psychotherapy and behavioral interventions: a meta-analysis. Behavior Modification.

[ref-27] Liu J, Zheng X, Chai S, Lei M, Feng Z, Zhang X, Lopez V (2018). Effects of using WeChat-assisted perioperative care instructions for parents of pediatric patients undergoing day surgery for herniorrhaphy. Patient Education and Counseling.

[ref-28] Liu NH, Daumit GL, Dua T, Aquila R, Charlson F, Cuijpers P, Druss B, Dudek K, Freeman M, Fujii C, Gaebel W, Hegerl U, Levav I, Munk Laursen T, Ma H, Maj M, Elena Medina-Mora M, Nordentoft M, Prabhakaran D, Pratt K, Prince M, Rangaswamy T, Shiers D, Susser E, Thornicroft G, Wahlbeck K, Fekadu Wassie A, Whiteford H, Saxena S (2017). Excess mortality in persons with severe mental disorders: a multilevel intervention framework and priorities for clinical practice, policy and research agendas. World Psychiatry.

[ref-29] Ma H (2012). Integration of hospital and community services-the ‘686 Project’-is a crucial component in the reform of China’s mental health services. Shanghai Arch Psychiatry.

[ref-30] McGuire AB, Bartholomew T, Anderson AI, Bauer SM, McGrew JH, White DA, Luther L, Rollins A, Pereira A, Salyers MP (2016). Illness management and recovery in community practice. Psychiatric Rehabilitation Journal.

[ref-31] Miller BJ, Stewart A, Schrimsher J, Peeples D, Buckley PF (2015). How connected are people with schizophrenia? Cell phone, computer, email, and social media use. Psychiatry Research.

[ref-32] Mojtabai R, Fochtmann L, Chang SW, Kotov R, Craig TJ, Bromet E (2009). Unmet need for mental health care in schizophrenia: an overview of literature and new data from a first-admission study. Schizophrenia Bulletin.

[ref-33] Naslund JA, Aschbrenner KA (2019). Risks to privacy with use of social media: understanding the views of social media users with serious mental illness. Psychiatric Services.

[ref-34] Naslund JA, Aschbrenner KA, Bartels SJ (2016). How people with serious mental illness use smartphones, mobile apps, and social media. Psychiatric Rehabilitation Journal.

[ref-35] Oksuz E, Karaca S, Ozaltin G, Ates MA (2017). The effects of psychoeducation on the expressed emotion and family functioning of the family members in first-episode schizophrenia. Community Mental Health Journal.

[ref-36] Pang H (2018). Is mobile app a new political discussion platform? An empirical study of the effect of WeChat use on college students’ political discussion and political efficacy. PLOS ONE.

[ref-37] Pekkala E, Merinder L (2002). Psychoeducation for schizophrenia. Cochrane Database of Systematic Reviews.

[ref-38] Sahu M, Grover A, Joshi A (2014). Role of mobile phone technology in health education in Asian and African countries: a systematic review. International Journal of Electronic Healthcare.

[ref-39] Salzmann-Erikson M, Sjodin M (2018). A narrative meta-synthesis of how people with schizophrenia experience facilitators and barriers in using antipsychotic medication: implications for healthcare professionals. International Journal of Nursing Studies.

[ref-40] Shafer A (2005). Meta-analysis of the brief psychiatric rating scale factor structure. Psychological Assessment.

[ref-41] Sheehan DV, Harnett-Sheehan K, Raj BA (1996). The measurement of disability. International Clinical Psychopharmacology.

[ref-42] Sorrell JM (2014). Moving beyond caregiver burden: identifying helpful interventions for family caregivers. Journal of Psychosocial Nursing and Mental Health Services.

[ref-43] Torous J, Keshavan M (2016). The role of social media in schizophrenia: evaluating risks, benefits, and potential. Current Opinion in Psychiatry.

[ref-44] Villani M, Kovess-Masfety V (2017). How do people experiencing schizophrenia spectrum disorders or other psychotic disorders use the internet to get information on their mental health? literature review and recommendations. JMIR Mental Health.

[ref-45] Wang SL, Wang Q, Yao J, Zhao SB, Wang LS, Li ZS, Bai Y (2019). Effect of WeChat and short message service on bowel preparation: an endoscopist-blinded, randomized controlled trial. European Journal of Gastroenterology & Hepatology.

[ref-46] WHO (2017). WHO disability assessment schedule 2.0 (WHODAS2.0). http://www.who.int/classifications/icf/whodasii/en/.

[ref-47] World Health Organization (2010). Schizophrenia. Retrieved on 15 April 2019. http://www.who.int/mental_health/management/schizophrenia/en/.

[ref-48] Zhang X, Wen D, Liang J, Lei J (2017). How the public uses social media wechat to obtain health information in china: a survey study. BMC Medical Informatics and Decision Making.

