# Supplementary material for: WeChat-based mHealth intention and preferences among people living with schizophrenia
Source: PeerJ. 2020 Dec 16;8:e10550. doi: 10.7717/peerj.10550 (PMC7749651; doi:10.7717/peerj.10550)
Supplement: Supplemental Information 2 [file peerj-08-10550-s002.docx]

******************

***paper4 preference for WeChat-based mHealth

******************

***disposal

gen wintention=1 if wpsy==1 |wpeer==1|wprof==1

replace wintention=0 if wintention==.

gen nintention=wpsy+wpeer+wprof

***Table 1. Preferences for WeChat-based interventions

tab1 wpsy wpeer wprof wintention nintention, missing

***Table 2. Social-demographic characteristics of PLS who did or did not support WeChat-based health interventions (N=400)

***total

tab1 agecat1 sex marriage1 education1 work1, missing

tab agecat1 wintention, chi column

tab sex wintention, chi column

tab marriage1 wintention , chi column

tab education1 wintention, chi column

tab work1 wintention, chi column

sum BST GAF tdiasbility

ttest BST, by(wintention)

ttest GAF, by(wintention)

ttest tdiasbility, by(wintention)

***Table 3. predictor of Preferences for WeChat-based interventions

logistic wintention i.agecat1 i.sex i.marriage1 i.education1 i.work1

logistic wintention i.agecat1 i.sex i.marriage1 i.education1 i.work1 BST GAF tdiasbility
